# Impact of metabolic syndrome on the progression of arterial stiffness in people of African ancestry

**DOI:** 10.4314/ahs.v26i1.14

**Published:** 2026-03

**Authors:** Omotayo Alaba Eluwole, Adeleye Adeomi, Kgothathso Nkoana, Muzi Joseph Maseko

**Affiliations:** 1 Department of Medical Pharmacology and Therapeutics, Faculty of Basic Clinical Sciences, Obafemi Awolowo University, Ile-Ife, Nigeria; 2 Department of Community Health, Faculty of Clinical Sciences, Obafemi Awolowo University. Ile-Ife, Nigeria; 3 School of Physiology, Faculty of Health Sciences, University of the Witwatersrand, Johannesburg, South Africa

**Keywords:** Metabolic Syndrome, Arterial Stiffness, Pulse Wave Velocity

## Abstract

**Background:**

Metabolic syndrome (MS) is an atherogenic risk factor influenced by both modifiable and non-modifiable risk factors, including race, hypertension, obesity, and age. This study evaluated the association between MS and arterial stiffness (AS) in individuals of African ancestry.

**Methods:**

Using WHO criteria, MS was assessed in 668 participants aged 18-70 years. Obesity was evaluated through body mass index (BMI), waist circumference (WC), and waist-to-hip ratio (WHR). Blood pressure (BP) measurements included office BP, 24-hour ambulatory BP monitoring (ABPM), and daytime/nighttime BP. Arterial stiffness was assessed via pulse wave velocity (PWV). Blood samples were analyzed for triglycerides (TG), high-density lipoprotein (HDL), and fasting blood glucose. Statistical analysis was performed using SPSS and STATA.

**Results:**

The prevalence of metabolic syndrome increased with age and was significantly higher in females. Participants with MS had a higher prevalence of hypertension and obesity. PWV was significantly associated with BP parameters and obesity indices (BMI, WC, WHR). Moreover, PWV was higher in individuals with MS compared to those without.

**Conclusion:**

Obesity and hypertension, key diagnostic components of MS, are independently associated with arterial stiffness. This underscores their role in driving target organ damage among individuals with MS in this African ancestry cohort.

## Introduction

World Health Organization emphasised five major components of metabolic syndrome, which include obesity, hypertension, insulin resistance, dyslipidaemia, and microalbuminuria^1^,^2^. Central or abdominal obesity is the first criterion attributed to metabolic syndrome (MS), simultaneously, it is also a risk factor for other components of MS^1^,^2^. Crude assessment of anthropometric measures of abdominal obesity, such as body mass index (BMI), waist circumference (WC), and waist-hip ratio (WHR) are strong and consistent predictor of cardiovascular morbidity^3^,^4^. Several studies have demonstrated that all components of MS are independently associated with arterial stiffness (AS)^5^-^7^. Notably, the accuracy of the association is dependent on the component of MS and the ethnicity of the participants^6^,^7^. However, uncontrolled hypertension and obesity are atherosclerotic risk factors strongly associated with vascular remodeling and hardening of the inner layers of the aorta and other large arteries, known as AS. Arterial stiffness, is the hallmark of atherosclerosis^5^ a leading contributor to the rising incidence of cardiovascular complications of MS. It is the macroscopic manifestation of endothelia dysfunction that involves several signaling pathways such as renin aldosterone angiotensin (RAS), reactive oxidative stress (ROS), insulin resistance (IR) and metabolism of advanced glycation end (AGE) products^5^,^6^.

Aortic pulse wave velocity (PWV) is the gold standard for the assessment of AS. It is usually measured between the carotid and femoral artery. It is directly proportional to arterial wall width and the measurement of elastin and collagen concentration, or AS, and it is inversely proportional to vessel diameter and blood viscosity^8^. Hence, higher arterial wall width and stiffness may result in higher PWV. However, the accuracy of the association depends on the component of MS and the ethnicity^7^. This is associated with endothelial dysfunction in physological and pathological conditions such as aging, MS, hypertension, and obesity^9^. Notably, PWV increases proportionally to the number of cardiovascular risk factors^9^. Hence, our study investigated the association between assessment of AS (PWV), obesity, and hypertension among Africans with a high prevalence of the two major diagnostic criteria.

## Methods

### Study design and population

Participants were stratified according to WHO criteria for MS. Out of the 1516 participants, 668 fulfilled the stipulated requirements. Participants were provided with information sheets detailing the purpose and process of the study. Each participant gave written informed consent for his/her voluntary participation in the study.

Ethical approval was obtained from the University of Witwatersrand, Human Research Ethics Committee [Medical (Reference number: M190472)].

### Measurements and Biochemical analysis

#### i. Anthropometric measurement

Anthropometric measurement was assessed according to the methods described by Eluwole et al., 2022 [10].

#### ii. Conventional (clinic) blood pressure assessment

Conventional blood pressure was measured using an automated sphygmomanometer (Omron, Kyoto, Japan) after 10 min of rest in the seated position. A regular adult cuff of 12 cm wide and 30 cm long was used for arm circumferences less than 33 cm, while a large adult cuff of 16 cm wide and 36 cm long was used for arm circumference that is greater than 33 cm^10^. Brachial blood pressure was recorded to the nearest 2 mmHg. Korotkov phases I and V were identified as systolic blood pressure (SBP) and diastolic blood pressure (DBP), respectively. Five consecutive BP readings were obtained. The average of the five readings was taken as the BP. Participants were classified as hypertensive if the mean value of blood pressure was higher than 140/90 mmHg (WHO standard for MS).

#### iii. Ambulatory blood pressure monitoring

Ambulatory 24-hr day and night BP was determined using SpaceLabs monitors (model 90207; Spacelabs, Redmond, WA). The cuff size is the same as that used in conventional BP measurements. Monitors was programmed to measure 24-hr BP at 15 minutes intervals during the day time (6:00 - 22:00) and at 30 minutes interval during the night time (22:00 - 06:00). Fixed clock periods as opposed to actual in-bed and out of bed periods were statistically analysed to ensure that similar day and night periods was selected for comparison among individuals^10^,^11^,^12^.

#### iv. Pulse wave measurement and analysis

Aortic pulse wave velocity was determined using applanation tonometry and SphygmoCor software^11^. The participants rested for 15 minutes in a supine position. Arterial waveforms with an electrocardiogram recording were recorded at carotid-femoral pulse simultaneously by applanation tonometry during 8-second period using a high-fidelity SPC-301 micromanometer (Millar Instrument, Houston, TX) interfaced with a computer employing SphygmoCor, version 6.21 software AtCor, Medical, West Ryde, New South Wales, Australia). Recordings were discarded when the systolic or diastolic variability of consecutive waveforms exceeds 5% or when the amplitude of the pulse wave signal is < 80 mV. The time delay in the pulse wave between the carotid and femoral sites was determined using the R wave of electrocardiograph recordings as a fiducial point. Pulse transit time was taken as the average of 10 consecutive beats. Distance from the suprasternal notch to the carotid sampling site (distance A) and from the suprasternal notch to the femoral artery (distance B) was measured. Pulse wave velocity was calculated as distance B minus distance A. Aortic PWV was calculated as distance (meters) divided by transit time^12^,^13^.

#### v. Biochemical analysis

After an overnight fast, a 5ml of venous blood sample was collected from the cubital fossa using an aseptic procedure. Lipid profiles [Triglyceride (TG), low-density lipoprotein (LDL), High-density lipoprotein (HDL), and fasting blood sugar were analysed at Contact Laboratory Services (CLS).

#### vi. Diagnosis of metabolic syndrome

Metabolic syndrome was defined according to WHO criteria^1^; insulin resistance was defined as type 2 diabetes mellitus (DM) or impaired fasting glucose (IFG) (> 100 mg/dl) or impaired glucose tolerance (IGT), plus two of the following:

Abdominal obesity (waist-to-hip (WHR) ratio > 0.9 in men or > 0.85 in women, or body mass index (BMI) ≥ 30 kg/m2.
Triglycerides 150 mg/dl or greater, and/or high-density lipoprotein (HDL)-cholesterol < 40 mg/dl in men and < 50 mg/dl in women.
Hypertension; 140/90 mmHg or greater.
Microalbuminuria (urinary albumin secretion rate 20 µg/min or greater, or albumin-to-creatinine ratio 30 mg/g or greater).

### Statistical analysis

All analyses were conducted using SPSS software for Windows, version 11.0J (SPSS, Chicago, USA) and STATA. P value of <0.05 was considered to denote statistical significance. Continuous data were reported as mean ± SEM. Results from the participants were compared between the two groups using Student's t-test. The χ2 statistic was used to compare means and proportions. Stata/MP 16.0 (StataCorp, College Station, TX, USA) was used to analyse the association between MS and different assessments of BP. Multiple regression and Pearson's correlation coefficient were used to determine the association between MS status and changes in peripheral BP, central BP, and ABPM. P value < 0.05 was considered significant. All models were also adjusted for all the assessments of PWV.

## Results

Table 1 shows the general characteristics of the study population according to MS status using WHO criteria. Hypertension and diabetes were significantly higher in those with metabolic syndrome. Participants with MS were older than those without metabolic syndrome. Lifestyle factors, smoking, and alcohol intake were not significantly different between the two groups.

**Table 1:** General characteristics of the study population according to MS status (WHO criteria)

value	Total population	Without MS	With MS	P
Number	668	588	80	
Age				
(years)	48.5±18.1	42.4±17.9	56.2±14.4	
	0.0200*			
Female				
(%)	61.7	59.7	76.2	
	0.0030*			
Alcohol				intake
(%)	19.3	18.9	22.5	
	0.2611			
Smokers				
(%)	16.6	17 �	13.8	
	0.2887			
Hypertensive				
(%)	45.7	40.6	82.5	
	<0.0001*			
Diabetic				
(%)	9.6	3.9	51.2	
	<0.0001*			

MS- Metabolic syndrome

* P value <0.05 depicts significant difference between those with and without MS

Table 2 shows haemodynamic characteristics of the study population according to MS status. The result revealed that SBP24, DBP24, SBPN, DBPN,SBPD, DBPD, SBPC, DBPC, DBPC were significantly higher in those with metabolic syndrome compared to those without metabolic syndrome. SBP24, 24-hoursystolic BP; SBPD, 24-hour diastolic BP; SBPN, night-time systolic BP; DBPN, night-time diastolic BP; SBPD,daytime systolic BP; DBPD, daytime diastolic BP; SBPC, conventional systolic BP; DBPC, conventional diastolic BP; C_SBP, centralsystolic BP; C_DBP, centraldiastolic BP. *p-value < 0.05 was considered significant. values expressed as mean ±standard deviation; p value < 0.05 considered significant.

**Table 2:** Haemodynamic characteristics of the study population (mm Hg) according to MS status

Total population	Without MS	With MS	P value
SBP24	662	117.1 ± 14.1	125.4
±17.8	<0.001*		
DBP24	662	72.2 ± 9.6	76.0 ±
11.0	0.100		
SBPN	662	110.4 ±16.2	119
±21.0	0.011*		
DBPN	662	64 ± 11.2	68.6 ±
12.7	0.020 *		
SBPD	662	121.3 ± 13.7	128.5 ±
16.4	0.001*		
DBPD	662	77.1 ± 9.5	80.5 ±
10.6	0.170		
SBPC	688	127.2 ± 20.9	140.1 ±
24.1	0.005*		
DBPC	688	82.6 ±11.5	88.4
±14.9	0.001*		
C_SBP	647	117.7 ±23.5	126.6
±22.6	0.002*		
C_DBP	647	83.32 ± 12.9	87.5 ±
11.4	0.006*		

Table 3 shows the association between MS and BP in the WHO category of MS. The result revealed that SBP24, DBP24, SBPN, DBPN, SBPD, DBPD, SBPC, and DBPC were significantly associated with PWV. CI, confidence intervals, SBPC- Conventional Systolic Blood Pressure, DBPC- Conventional Diastolic Blood Pressure, SBP 24 - 24-hour Systolic Blood Pressure, DBP24 - 24-hour Diastolic Blood Pressure, SBPD-Daytime Systolic Blood Pressure, DBPD- Daytime Diastolic Blood Pressure, SBPN - Nighttime Systolic Blood Pressure, DBPN - Nighttime Diastolic Blood Pressure, PWV- pulse wave velocity, *means there was a significant p value when compared with metabolic syndrome category, p < 0.05. Values expressed as a percentage or mean ±standard deviation; p value < 0.05 considered significant.

**Table 3:** The association between MS and BP (mm Hg) in the WHO

	R^2^	CI
	P value	
PWV vs		
SBP24	0.171	0.061 to 0.091
	< 0.001*	
DBP24	0.097	0.062 to 0.110
	< 0.001*	
SBPN	0.163	0.051 to 0.077
	< 0.001*	
DBPN	0.109	0.058 to 0.099
	< 0.001*	
SBPD	0.142	0.056 to 0.088
	< 0.001*	
DBPD	0.064	0.046 to 0.095
	< 0.001*	
SBPC	0.303	0.059 to 0.074

Table 4 shows the multivariate association between PWV and the indices of obesity. The analysis shows that the three indices of obesity (WC, BMI, and WHR) were significantly associated with PWV. PWV- Pulse waist velocity, WC- waist circumference, BMI-body mass index. Corrected for age, sex, smoking, and alcohol intake; CI, confidence intervals; values expressed as a mean ±standard deviation; p value < 0.05 considered significant.

**Table 4:** Multivariate association between PWV and the indices of obesity

		R^2^	CI
	P value		
PWV vs			
WW (cm)		0.090	0.038 to 0.062
	< 0.001*		
BMI (kg/m^2^)		0.043	
	<0.001*		
WHR		0.054	2.754 to 5.338
	<0.001*		

## Discussion

**Fig 1:**
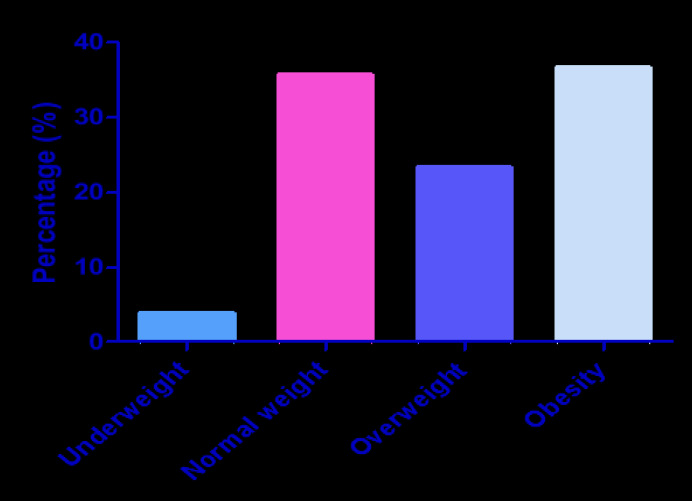
In the study population, 4% were underweight, 35.8% had normal weight, 23.4% and 36.8% were overweight and obese, respectively

**Fig 2:**
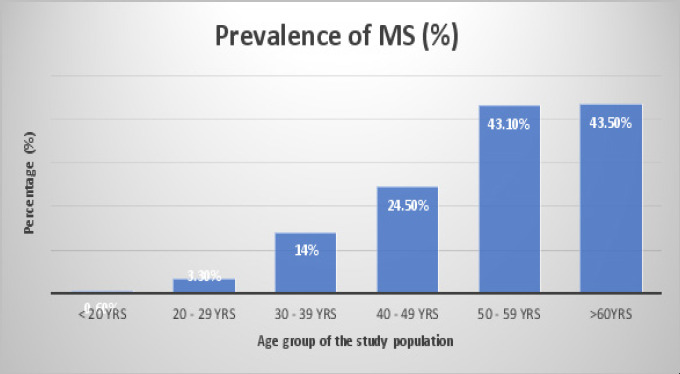
Showing the prevalence of metabolic syndrome (MS) among the age groups in the study population

Our study revealed a high prevalence of obesity (36.8%) among the study population [Fig. 1]. Metabolic syndrome (MS) was noticed to be common among female participants than male. Moreover, those with MS are significantly older than those without MS (Table 1). However, studies have shown that indices of obesity (WC, BMI, and WHR) and hypertension increase with increasing age^4^,^14^. Hence, the observed age-related increase in MS in this study (Fig. 2) was believed to be mediated by the indices of obesity and hypertension. Interestingly, two of the three indices (WC and BMI) and hypertension have been documented to be strongly related to insulin resistance^4^,^15^. Notably, the prevalence of diabetic participants in this study significantly increased among those with MS [Table 1].

According to the 2021 USPSTF hypertension screening recommendation, hypertension is defined^17^ as blood pressure >= 130/80 mm Hg. Our result implies that, irrespective of the WHO definition of hypertension, MS may be diagnosed among apparently healthy people with normal BP and isolated systolic hypertension (Table 2). Isolated systolic hypertension means systolic BP increases despite normal diastolic BP. Studies have shown that this is more prevalent in older adults and related to arterial stiffness and MS^18^; there is are risk of atherosclerosis^19^,^20^. As earlier stated, most participants with MS are hypertensive (82.5%) and older than those without MS (Table 1). Furthermore, in this study, there was an association between PWV and the haemodynamic parameters (Table 2). Likewise, all the BP measurements significantly correlate with PWV (Table 3). This corroborates studies by Pietri et al., and Omboni et al, 2019, which observed increased peripheral wave reflections and endothelial dysfunction in untreated essential hypertensive patients and suggested that ambulatory pulse wave analysis may help evaluate vascular health of individuals at risk for cardiovascular disease^21^,^22^.

Variability in ethnicity, gender, and other related components of MS are interrelated factors to the development of AS^23^-^25^. These factors influence the interwoven mechanisms involved in the development/progression of AS regardless of the contributory factors^24^,^25^. Regarding the gender differences, a study in Korean men revealed an association between AS in men and no association AS in women based on flexibility^25^. In addition, relative risk factors such as alcohol and smoking are also associated with accelerated progression of arterial stiffening^26^. Our findings show that the two risk factors were not significantly different between those with or without MS. On the contrary, Wang et al. (2022) reported that active smoking is associated with increased prevalence of MS, especially among those less than 70 years^27^. Yu et al. (2014) also documented that smoking and alcohol contribute to an increase in the prevalence of MS in Chinese men only^28^. However, the main focus of our study is on the impact of pathological conditions on the pathogenesis of AS. Therefore, obesity, persistent hypertension, and MS revealed in our study most likely result to distending pressure in the large elastic-type arteries (aorta and carotid) are key determinants of the degenerative changes; thus, our results show that PWV is significantly higher in individuals with MS compared to those without MS (Fig 1). This could be further linked with the pressure wave generated by the left ventricle, which travels down the arterial tree and then reflected at multiple peripheral sites, mainly at resistance arteries (small muscular arteries and arterioles). Consequently, the pressure waveform recorded at any site of the arterial tree is the sum of the forward traveling waveform generated by left ventricular ejection and the backward traveling wave, the pattern of the incident wave reflected at peripheral sites^30^,^31^. If the large conduit arteries are healthy and stable, the reflected wave merges with the incident in the proximal aorta during diastole, thus, augmenting the diastolic BP and enhance coronary perfusion. In contrast, if the arteries are hardened, pulse wave velocity increases, accelerating the incident and reflected waves; thus, the reflected wave merges with the incident wave in systole and augments aortic systolic rather than diastolic pressure^12^,^13^,^32^. As a result, left ventricular afterload increases and normal ventricular relaxation and coronary filling are compromised. Apart from changes in the timing of the waveforms merging, changes in the magnitude of the reflected wave and central pressures may result from changes in the proportion of the incident wave that is reflected^13^. Hence, the association between all the indices of obesity and AS (Table 4) implies that prevention/treatment of obesity and hypertension in Blacks will reduce the prevalence of MS and attenuate the progression of arterial damage and its associated complications.

## Conclusion

Obesity and hypertension are major components of MS emphasized by the WHO criteria; the two components are rampant among Africans. Our study observed that these two major factors remain important components in the determinants of the degenerative vascular changes among Africans. Therefore, interventions aimed at reducing the progression of arterial damage and associated complications should be targeted at the prevention and management of obesity and hypertension.

## Figures and Tables

**Figure 3:**
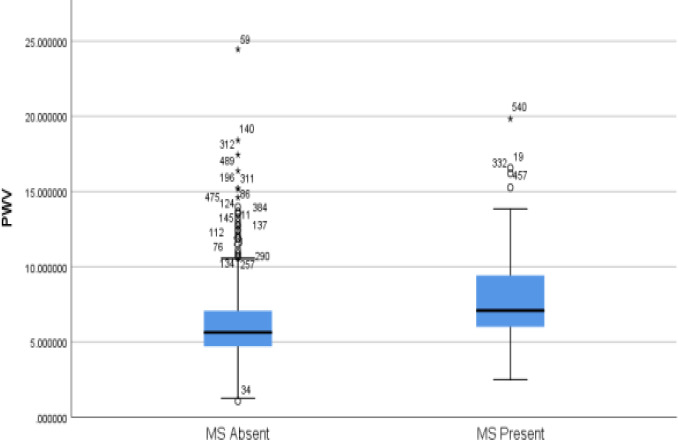
Shows the differences in PWV in the WHO category of MS. The PWV of participants with metabolic syndrome was significantly higher than participants without metabolic syndrome
